# Premembering Experience: A Hierarchy of Time-Scales for Proactive Attention

**DOI:** 10.1016/j.neuron.2019.08.030

**Published:** 2019-10-09

**Authors:** Anna C. Nobre, Mark G. Stokes

**Affiliations:** 1 Department of Experimental Psychology, University of Oxford, Oxford, UK; 2 Oxford Centre for Human Brain Activity, Wellcome Centre for Integrative Neuroimaging, Department of Psychiatry, University of Oxford, Oxford, UK

**Keywords:** memory, attention, decision-making, hippocampus, prefrontal cortex, priming, working memory, episodic memory, implicit memory

## Abstract

Memories are about the past, but they serve the future. Memory research often emphasizes the former aspect: focusing on the functions that re-constitute (re-member) experience and elucidating the various types of memories and their interrelations, timescales, and neural bases. Here we highlight the prospective nature of memory in guiding selective attention, focusing on functions that use previous experience to anticipate the relevant events about to unfold—to “premember” experience. Memories of various types and timescales play a fundamental role in guiding perception and performance adaptively, proactively, and dynamically. Consonant with this perspective, memories are often recorded according to expected future demands. Using working memory as an example, we consider how mnemonic content is selected and represented for future use. This perspective moves away from the traditional representational account of memory toward a functional account in which forward-looking memory traces are informationally and computationally tuned for interacting with incoming sensory signals to guide adaptive behavior.

## Teaser

Nobre and Stokes consider how memories guide proactive attention. Introducing the concept of premembering, this perspective advances a functional account of memory. Memory traces are informationally and computationally tuned for interacting with incoming sensory signals to guide adaptive behavior.

## Main Text

Memory’s most compelling illusion is that it represents the past. However, it is clear from an ecological perspective that memory is all about the future. The purpose of memory is to learn about the environment to anticipate future demands—not just putting back the pieces of the past for recollection (remembering experience) but deriving possibilities based on the past to guide future adaptive behavior (premembering experience). This notion has old roots (Helmholtz, 1867), but, surprisingly, the fundamental operations through which memories guide adaptive behavior lack an established theoretical framework, and their mechanisms have yet to capture the in-depth and systematic investigation they deserve. We propose that memory, over a broad hierarchy of timescales, supplies the essential elements for selective attention to guide perception and performance flexibly and adaptively. We introduce the term “premembering” to capture this prospective and dynamic role of memory. Our construct complements proposals for how memories can be used to inform other cognitive functions, such as adaptive control, decision-making, and imagining future situations (Box 1).

BOX 1 **Prospective Memories in Different Cognitive Domains**

We focus on how memories guide selective attention. The role memory plays in guiding behavior has also been considered within other cognitive domains.

“Cognitive control” refers to the collection of mechanisms that set and adjust our goals and that monitor and regulate performance according to competing demands. Thus, the selective attention mechanisms we review are subordinate to cognitive control, prioritizing and selecting putative targets and overcoming irrelevant distraction within a particular goal setting. Memories are increasingly recognized to play an important role in influencing the degree of top-down control exerted on a given trial. They include both short-term traces between successive trials as well as intermediate memory traces that develop over task performance (Chiu and Egner, 2019).
“Decision-making” refers to the process of choosing one of a set of alternatives to produce a beneficial outcome. Choices are made based on expectation of rewards developed through previous experience. Current models of decision-making mostly rely on reinforcement learning. Recent computational studies suggest that different types of memory traces may work together to optimize reinforcement learning, including those resulting from slow incremental implicit learning over trials as well as episodic traces uniquely linked to individual experiences (Botvinick et al., 2019).
“Decision-making” is closely related to selective attention, which can be involved in prioritizing information for guiding choice behavior. Expectations play an essential role in both sets of processes. However, interestingly, they each stress a different consequence of prior knowledge. In decision-making, predictions based on priors are mainly used to attenuate the processing of what can be anticipated (Friston, 2010). In selective attention, predictions are mainly used to enhance the processing of anticipated task-relevant information. These two phenomena nicely illustrate the flexibility with which memory-related traces can be used to guide adaptive performance. The specific consequence of prior knowledge will be heavily dependent on the purpose of the task (Nobre, 2018).
“Episodic future thinking” involves drawing on previous experiences to imagine oneself in future situations (Atance and O’Neill, 2001, Schacter et al., 2008). The construct is useful in different types of situations, such as navigation, planning to implement intentions, understanding others’ mental states, and simulating future events. Neuropsychological, developmental, and brain imaging studies have revealed substantial overlap between the neural system supporting episodic future thinking and episodic recollection, suggesting that the LTM traces available for recollection can also be used prospectively and flexibly to build novel plausible scenarios and run simulations (Schacter et al., 2008). Episodic future thinking differs from our construct of premembering in being a specifically deliberative process based on LTM traces available to awareness to inform behavior in the future. Premembering is a broader construct, considering the influences of memories of different types and timescales on ongoing or imminent behavior.

Selective attention (hereafter called “attention”) refers to the set of functions that prioritize and select information to guide adaptive behavior (Nobre, 2018). These functions modulate incoming sensory signals and influence their processing at multiple stages to inform awareness, decisions, actions, and subsequent memories. It has long been appreciated that short-term memory, or “working memory” (WM), plays an important role in forming attentional templates (e.g., Desimone and Duncan, 1995). Here we suggest that WM is part of a much larger family of heterogenous attention-guiding memory traces that span multiple timescales.

The premembering perspective has important implications for understanding the content and formatting of memory. Rather than slavishly storing and using veridical traces, the brain flexibly selects and even distorts memory content to enhance its utility in guiding attention. Moreover, task-relevant informational content may also be stored in a neural format that is optimized for the anticipated utilization of the memory to guide performance.

### Multiple Timescales of Memories Guide Attention

**Figure 1:**
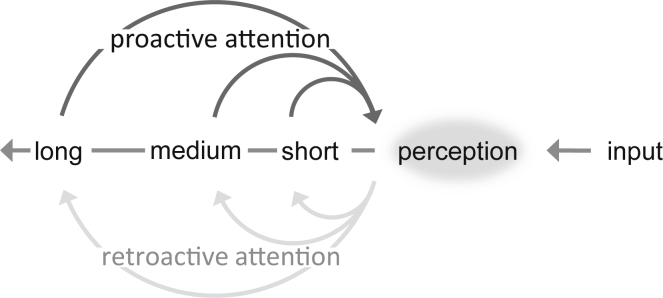
Mutual Interactions between Memory and Attention Attention draws on past experience from multiple timescales to anticipate and prepare for incoming stimulation and guide adaptive action. Conversely, attention is not only forward looking but can select and bias information during encoding and maintenance in memory. These mutual interactions feed a virtuous cycle that tunes our minds to the most relevant features of the environment. In this review, we consider the multiple mnemonic timescales that are important for guiding proactive attention (dark arrows).

The essence of memory is the traces left by passing experience. These range from transient perturbations to engrams that last a lifetime, capturing modality-specific fragments to relational and integrated wholes and supporting unconscious states to recollective phenomena. Such traces can provide essential informational content required for prospectively prioritizing and selecting what is important. Working together, memory content and attention functions shape how the brain transforms incoming signals to guide perception, choice, action, and the formation of new memories to serve adaptive behavior in the future (Figure 1). It can be argued that these prospective properties of memory are what define its fundamental ecological purpose: to collect relevant aspects of experience to anticipate future demands and guide behavior.

#### Short-Term Traces

**Figure 2:**
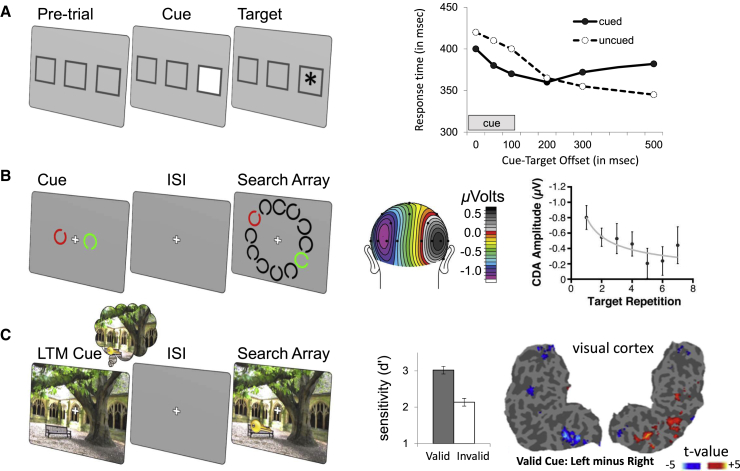
Multiple Timescales for Memory-Guided Attention (A) Even at very short time-scales, attention is influenced by preceding stimuli. For example, the classic exogenous cueing paradigm demonstrates how attention lingers at a previously cued location (adapted from Posner and Cohen, 1984). (B) At slightly longer timescales, WM guides visual search (quantified as contralateral delay activity [CDA]). However, as the timescale increases (over trials with repeating template), attentional control is transferred to intermediate memory (i.e., reduced CDA; scale bar represents relative voltage difference over the scalp surface; error bars represent ±1 SEM). Adapted from Carlisle et al. (2011). (C) At even longer timescales, LTM maintains relevant information for guiding attention. In this study, Stokes et al. (2012) found that spatial information stored in LTM can be used to modulate the visual cortex in preparation for a target stimulus (error bars represent ±1 SEM; scale bar represents the relative BOLD response in left and right visual cortex).

Drawing a clear line defining when present becomes past may be impossible. Nevertheless, it is clear that past traces affect perception even from their earliest moments. The very stitching of visual perception across eye movements into an apparent cohesive flow may rely on short-term memories bridging the anchoring and landing fixation contents (Irwin and Gordon, 1998). Furthermore, transient salient visual stimuli intrinsically capture attention and leave a brief excitatory trail, temporarily enhancing processing of stimuli that follow in their immediate wake (Posner, 1980; Figure 2A).

At slightly longer timeframes, WM provides a limited set of more durable traces that are independent of continuous sensory stimulation and resistant to interference and that act to guide adaptive behavior (Baddeley, 2003). The fundamental role WM plays in guiding attention is widely recognized and has been studied extensively (Desimone and Duncan, 1995). Information in WM has been considered the major source of top-down proactive attention. Even before the target stimuli appear, these memory traces influence the pattern of brain activity in a proactive fashion to facilitate the processing of signals associated with likely relevant items (Chelazzi et al., 1993, Kastner et al., 1999, Stokes et al., 2009). These top-down anticipatory states based on WM templates are often associated with willful, volitional orienting of attention (Helmholtz, 1867, Posner, 1980). However, it is important to note that WM content can also influence incoming information processing in an involuntary fashion (Soto et al., 2008; see below).

Moreover, beyond the scope of classic WM, the relation between successive items can also influence information processing. For example, when searching for an odd-one-out target, its identification is facilitated when the current target shares a defining feature value (Maljkovic and Nakayama, 1994) or location (Maljkovic and Nakayama, 1996) with the target in the preceding trial. Traces from preceding stimuli also alter the quality of perception. Reports of the spatial frequency and orientation of a visual item are systematically distorted by that of preceding items (e.g., Fischer and Whitney, 2014).

Short-term memory traces can be used to enhance or suppress information processing, depending on the situation. For example, after initial facilitation of stimuli in the wake of non-informative salient stimuli, decrements in performance occur at the same location; the “inhibition of return” has been proposed to encourage sampling and exploration (Posner and Cohen, 1984). Pre-exposure to irrelevant or distracting stimuli can facilitate performance in visual search tasks by helping overcome distractor interference (Olivers et al., 2006). However, when the current target shares the identity or features of a distractor in the previous trial, performance decrements occur (negative priming; Fanini et al., 2006, Tipper, 1985). Reward associations can further magnify both facilitatory and inhibitory effects between successive stimuli (Della Libera and Chelazzi, 2006, Kristjánsson et al., 2010).

#### Intermediate Traces

Although most research has focused on the role of short-term traces in anticipatory attention, the memory traces that guide our perception stretch further back in time. Most of the knowledge that guides the interpretation of sensory signals and sets up expectations about the occurrence and context of relevant events is stored in our long-term memory (LTM; Nobre and Mesulam, 2014). However, somewhere between short-term traces that guide performance between successive events and remote long-term memories that guide performance between distant episodes lives a rich variety of memory traces reflecting regularities learned and utilized within a task setting. An elegant study by Carlisle et al. (2011) showed the gradual transition from WM to a longer-term trace when search targets were held constant for a number of trials (Figure 2B). For early trials, they showed a robust event-related potential associated with the location and number of items in the WM template (contralateral delay activity [CDA]; Vogel and Machizawa, 2004). As trials progressed, the CDA progressively diminished, implying a gradual handover to a longer-term, intermediate memory store.

Increasing numbers of experimental approaches are beginning to explore how different types of intermediate traces guide attention within task settings. “Probability cueing” refers to performance benefits resulting from the higher prevalence of a task-relevant item within a given location (Shaw and Shaw, 1977). The frequent appearance of a target at a given location facilitates its identification, whereas the frequent appearance of an irrelevant stimulus at a location attenuates its distracting effect (Goschy et al., 2014, Wang and Theeuwes, 2018, Ferrante et al., 2018, Noonan et al., 2016). Memory traces linked to probability cueing are long lasting and largely acquired implicitly (Jiang 2018). A related but different phenomenon is “selection history” (e.g., Kyllingsbaek et al., 2001), which refers to facilitation in identifying stimuli previously selected as targets independent of their probability.

“Reward history,” referring to the likelihood or value of a reward typically associated with given target stimuli within a task, has strong and persistent effects on performance (Anderson et al., 2011, Chelazzi et al., 2014, Krebs et al., 2011, Raymond and O’Brien, 2009). Reward manipulations often come bundled with manipulations of other sources of attention modulation, such as probability cueing or selection history. In complex search environments, reward history combines with other factors to guide effective performance (e.g., Navalpakkam et al., 2010). In tasks that carefully control for other sources of influence, stimuli with features that have high reward associations act as potent distractors, even when they are otherwise inconspicuous and task irrelevant (Failing and Theeuwes, 2015, Hickey and Peelen, 2015).

Learning and utilization of more complex associations also occur within task settings. “Contextual cueing” shows that pick-up of repeated spatial configurations of stimuli within visual search arrays facilitates target identification (Chun and Jiang, 1998). This type of memory guides search behavior, resulting in fewer fixations and faster search times (Harris and Remington, 2017). Neurophysiological markers of target detection are also enhanced (Johnson et al., 2007, MacLean and Giesbrecht, 2015, Schankin and Schubö, 2009). Learning of statistical regularities over time similarly leads to improvements in behavioral performance (Schapiro et al., 2016). Interestingly, the presence of statistical regularities within stimulus streams spontaneously captures attention, leading to facilitation of target identification in structured relative to unstructured streams (Zhao et al., 2013). Both contextual cueing and statistical learning are thought to involve hippocampus-related memory systems, even though they are learned largely implicitly (Chun and Phelps, 1999, Schapiro et al., 2016). A recent neurophysiological study of sequence learning in a serial response task has also revealed proactive anticipation of upcoming stimuli and associated responses based on learned spatiotemporal expectations (Heideman et al., 2018).

#### Long-Term Traces

Although the role of LTM in guiding attention has been less well explored than that of WM, its fundamental role in defining perception, choosing actions, imagining, and forming new memories has long been recognized (Bartlett, 1932, Helmholtz, 1867, Moscovitch et al., 2016; Box 1). We propose that the role memory plays in guiding attention may contribute to its fundamental influence over our mental experience and behavior.

In everyday situations, the bank of remote memory traces from previous settings and episodes arguably provides the richest source of information for guiding attention and perception. When waiting to meet a friend on a busy street, we rely on LTM to search them out based on their appearance and likely route. LTM provides knowledge about whether and how relevant events occur in particular contexts and about their likely reward outcome values. Thus, they afford rich content for flexible, proactive, and dynamic biases to guide the prioritization and selection of relevant information (Aly and Turk-Browne, 2017, Bar, 2004, Nobre and Mesulam, 2014). An increasing variety of experimental approaches is being used to explore how LTM content is used proactively to guide adaptive behavior. Although differing in detail, the approaches share the notion that the brain uses LTM information constantly, proactively, and predictively.

In visual search tasks using simple stimulus arrays, semantic knowledge of features commonly associated with objects facilitates target identification, overcoming usual costs associated with feature binding (Rappaport et al., 2013). On the other hand, distractors with associative links to the target disrupt the visual search by capturing attention (Moores et al., 2003). Furthermore, newly learned associations continue to affect performance on subsequent tasks, even when completely irrelevant (Fan and Turk-Browne, 2016).

In tasks using complex scenes or environments, “contextual priming” studies show that previous experiences within particular contexts guide the identification and interpretation of objects within the same contexts (Bar, 2004). When searching for a specific item within a scene, LTM works alongside lower-level statistics of visual features to guide performance (Navalpakkam and Itti, 2006). Multiple types of LTM are at play (Wolfe et al., 2011, Le-Hoa Võ and Wolfe, 2015). Search performance benefits from schematic probabilistic knowledge of encountering the item within a particular context (Võ and Henderson, 2009) as well as from its likely location relative to the layout of the scene (Torralba et al., 2006) and to other objects within a scene (Bar, 2004). In addition, specific knowledge about the location of an item within a particular scene also contributes—episodic guidance (Brockmole and Henderson, 2006). Interestingly, incidental memories formed through active search and selection of items within a scene lead to stronger performance benefits and subsequent memories than familiarity or explicit attempts to memorize items within scenes (Draschkow et al., 2014), suggesting that effective memories fall out of natural behavioral interactions.

Memory-guided attention tasks show how learning of object locations through repeated visual search in complex scenes or environments facilitates performance in subsequent tasks. Performance measures reveal significant improvements in detecting and discriminating target items as a result of previously learned specific contextual association between the particular item and a scene or three-dimensional environment (Becker and Rasmussen, 2008, Draschkow and Võ, 2016, Kit et al., 2014, Summerfield et al., 2006).

“Memory-guided orienting” tasks provide a good platform to investigate anticipatory biases based on long-term memories (Summerfield et al., 2006; Figure 2C). Participants form object-scene associations to a high and stable level during a learning session; the consequences of these memory traces are then tested in a separate attention-orienting session. The separation helps disentangle effects of early learning from those of subsequent memory utilization. In our own research, we have used learned scenes presented in isolation as memory cues, followed by the appearance of a target stimulus to be identified or discriminated. The critical behavioral comparison is between performance depending on whether the target location is correctly predicted by previous memory experience (valid memory cue) versus incorrectly predicted (invalid memory cue) or unpredicted (neutral memory cue). Strong and reliable effects occur for both perceptual sensitivity (Patai et al., 2012) and response times (Summerfield et al., 2006). By presenting memory cues and targets individually, it is possible to measure memory-related brain activity that biases excitability according to anticipation of the target location. fMRI reveals that memory cues engage the hippocampus as well as the dorsal frontoparietal network associated with the control of spatial attention (Summerfield et al., 2006, Stokes et al., 2012). In addition, activity levels in visual cortical regions become spatially biased in anticipation of the target location (Stokes et al., 2012; Figure 2C). Electrophysiological recordings also reveal memory-related spatial anticipatory biases in the form of lateralization of alpha-band power (Stokes et al., 2012, Summerfield et al., 2011). Anticipatory biases in the cue-target period are followed by modulation of event-related potentials related to visual processing (Summerfield et al., 2011) and spatial selection (Patai et al., 2012, Doallo et al., 2013) of target items. Reward associations further potentiate the effects of memory-based orienting. Even a single association with a modest monetary gain or avoidance of loss during the last learning block leads to performance improvements in the subsequent attention-orienting session, even though reward is irrelevant to the orienting task. Target stimuli during the orienting task elicit larger visual P1 potentials when previously associated with reward or punishment avoidance (Doallo et al., 2013, Suárez-Suárez et al., 2019).

Rosen et al. (2014) have followed a similar approach involving learning and utilizing spatial contextual associations in a change-detection task. By comparing memory-guided and visually guided attention conditions, they have highlighted a set of cortical regions and differences in hemispheric lateralization associated with LTM-based orienting (Rosen et al., 2016).

Thus, findings with memory-guided orienting tasks clearly illustrate the ability of the brain to use LTM content proactively to guide prioritization and suggest the involvement of limbic, memory-related circuits. In monkeys, recent studies recording activity from neurons in the hippocampus and entorhinal cortex during free viewing provide converging evidence that medial temporal areas, traditionally associated with memory, are closely related to attention. Neurons in the entorhinal cortex show strong visuospatial coding properties, signaling gaze location using multiple frames of reference (Meister and Buffalo, 2018). Entorhinal neurons further show a grid cell-like arrangement of viewed spatial locations, using only covert attention in the absence of any eye or other physical movement (Wilming et al., 2018). In addition to grid cells, saccade direction cells are also present, with largely independent populations encoding the direction of previous versus future saccades (Killian and Buffalo, 2018). Together, such findings point to mechanisms that could support spatial attention based on LTM. Recordings in the hippocampus showed that slow-wave ripples, thought to promote plasticity, occurred during a search for object changes within scenes and that their probability increased with scene repetition and near remembered targets (Leonard and Hoffman, 2017). Such a mechanism would be a good candidate for supporting memory-guided attention during search.

#### Dynamic Prospective Memories

In addition to carrying information about the location and identifying attributes of anticipated events, memory traces also carry information about their timing. Memory-based temporal expectations can therefore enable the brain to prepare for events in a dynamic, temporally structured, and efficient way (Nobre and van Ede, 2018). Temporal expectations rely on learning temporal regularities that occur over multiple timescales. Neural markers of proactive temporal anticipation have been observed when the timing of stimuli follows a regular temporal rhythm (Cravo et al., 2013), probabilistic conditional probabilities (Cravo et al., 2011), or sequences (Heideman et al., 2018). Across episodes, long-term memories can also guide temporally structured anticipatory attention. Using a temporal variant of the memory-guided orienting task, Cravo et al., (2017) showed improved perceptual sensitivity and response times to detect and discriminate visual targets occurring at the learned temporal interval (valid memory cue) relative to the other interval (invalid memory cue). Event-related potentials elicited by the memory cue revealed clear modulation of target anticipation according to temporal expectation. Behavioral and neural markers of validity effects correlated with one another and with the quality of learning of the temporal association.

#### Plurality across Types and Timescales

Recognizing that memory traces of different types and timescales proactively guide perception invites us to reconsider dominant concepts and dichotomies in the attention literature. The simple separation between “bottom-up” sources of prioritization based on physical salience and “top-down” sources based on current goals is clearly insufficient. To explore the natural kinds of memory-based attention, it is important to separate two fundamental factors: their volitional character and their source.

Subjectively, attention can be voluntary or involuntary, and its source can be external, based on physical salience (exogenous), or internal, based on brain states linked to current goals or previous experience (endogenous). Often these two factors are conflated, with exogenous sources assumed to guide involuntary attention and endogenous sources assumed to guide voluntary attention. Instead, they should be viewed as theoretically separable. Although physically salient stimuli capture attention involuntarily, endogenous sources can guide attention both voluntarily and involuntarily. In addition to their use in goal-based voluntary attention, both short-term as well as LTM sources can also have automatic, involuntary consequences for perception. For example, short-term memory carrying goal-related information to perform one task also inadvertently influences performance in another task, sometimes causing significant interference (Soto et al., 2008). Memory traces acquired over a task setting—reflected in probability cuing, selection history, reward history, contextual cueing, or sequence learning—are often acquired incidentally and utilized involuntarily and without explicit knowledge (Chun, 2000, Jiang, 2018). Remote, long-term memories for the presence and location of items within rich, complex scenes are more often accompanied by conscious recollection (Brockmole and Henderson, 2006, Jiang, 2018). Although it is likely that these associations are used to direct attention voluntarily, they may also orient attention involuntarily. Under most conditions, benefits in LTM-based orienting correlate with measures of explicit memory (Cravo et al., 2017, Salvato et al., 2016), suggesting a viable source for voluntary attention. However, dissociations have also been noted. Although explicit retrieval of object-scene memories is significantly compromised in older individuals compared with younger counterparts, the benefits of memory-based attention are spared (Salvato et al., 2016). The relationship and the degree of interaction between these two modes of memory-based attention are important directions for future research (Nobre and Mesulam, 2014).

In recognizing the role that memory associations within task settings play in attention, some contemporary models of attention have proposed adding a third, “history” source to the traditional bottom-up and top-down sources in attention (e.g., Awh et al., 2012). The inclusion is an important first step toward acknowledging the fundamental role played by memory, but there are also inherent limitations with treating memory as a single and separate additional source of attention control. On one hand, it is not possible to extricate memory from the two traditional sources of attention. Brief memory traces contribute to effects of bottom-up attention, prioritizing stimuli that trail physical singletons, and WM traces are essential for goal-based top-down attention. On the other hand, amalgamating memory sources into one common history factor works against evidence suggesting the plurality of memory mechanisms at play.

The internal, memory-based sources of attention are many. Memories of various durations and types contribute, from traces that stitch the continuity of perception to those that guide the recognition of a long-lost friend. Most likely, multiple memory systems and mechanisms are involved. For example, both contextual cueing and memory-based orienting involve LTM for the location of a target object within a given configuration in an array or scene, and in both cases, the medial-temporal areas have been implicated. However, brain imaging shows increased hippocampal activity during memory-guided orienting in scenes (Hannula and Ranganath, 2009, Summerfield et al., 2006) and decreased activity by repeated versus novel arrays in contextual cueing tasks (Greene et al., 2007, Giesbrecht et al., 2013). Electrophysiological recordings also show different patterns of modulation of potentials associated with target selection (N2pc), with attenuation by memory-guided orienting (Doallo et al., 2013, Patai et al., 2012) and enhancement by contextual cueing (Kasper et al., 2015, Schankin and Schubö, 2009). Striking differences occur even when comparing different types of memory traces within a single task. Goldfarb et al., (2016) contrasted the effects of contextual cueing (associations between specific array configurations and target locations) versus probability cueing (probabilistic learning of most likely target locations and response choices) in guiding visual search in a common task. Although contextual cueing modulated activity in the hippocampus, probability cueing modulated activity in the striatum. The effects were selectively predictive of attention benefits across successive trials and correlated with performance measures. Numerous functional dissociations have also been observed in behavioral effects of attention based on different types of memory within a task context. Although often entangled in natural contexts, probability cueing effects can be separated from single-trial priming effects (Jiang 2018), from selection history (Ferrante et al., 2018, Wang and Theeuwes, 2018), and from goal-based, short-term-memory cueing (Goschy et al., 2014, Jiang, 2018). Likewise, reward history can be separated from selection history (Anderson et al., 2017).

Thus, the literature suggests a rich diversity of mnemonic influences on attention, with at least some functional dissociations. There is much empirical work ahead to reveal the natural kinds and neural mechanisms of memory-based attention to derive its principles of organization and develop useful theoretical models. Traces of different kinds and durations could act separately and largely independently; they could compete, they could become integrated within a unified predictive model (e.g., in a Bayesian framework; Friston, 2010), they could converge and operate through a common priority map to modulate sensory processing (Bisley and Goldberg, 2010), or they could combine in mutually supportive ways to enhance the quality and flexibility of biasing signals (Botvinick et al., 2019). At this stage, it is premature to clump together memories into a single source of attentional control, and doing so could discourage or misguide much needed investigation in this important area.

### Prospective and Adaptive Memories

Premembering has implications for understanding the nature of memory itself. Consonant with a role in serving future behavior by grounding attentional selection, the very nature of memory traces is forward looking. What is encoded, maintained, and selected for retrieval is strongly influenced by what is likely to be important for future behavior.

#### Future-Relevant Content of Short-Term Memory

At the shortest timescales, the contents of memories maintained across eye fixations are strongly influenced by the location of the upcoming saccade. Even before a saccade is made, the information corresponding to its upcoming landing zone is remembered better than information at other, equidistant locations (Irwin, 1992).

Within WM research, findings increasingly highlight the prospective qualities of these traces. The contents of WM are strongly influenced by current task goals. They are selective and can even be distorted to facilitate performance based on anticipated task demands. The selectivity of WM was elegantly illustrated by a neurophysiological study in monkeys showing that neuronal activity in the prefrontal cortex (PFC) was largely dominated by the single relevant item of a multi-item array during a selective delayed-match-to-sample task (Rainer et al., 1998). Later, responses in prefrontal neurons were shown to be most sensitive to stimulus dimensions that were relevant for making categorical discriminations imposed by the task (Freedman et al., 2001). Although prefrontal neurons differentiated these discriminant features, they were less sensitive to other equally available, non-discriminant physical features until these became task relevant. Similar effects were reported for the parietal cortex when monkeys performed categorical discrimination tasks based on arbitrary boundaries along continuous feature dimensions (Freedman and Assad, 2006).

**Figure 3:**
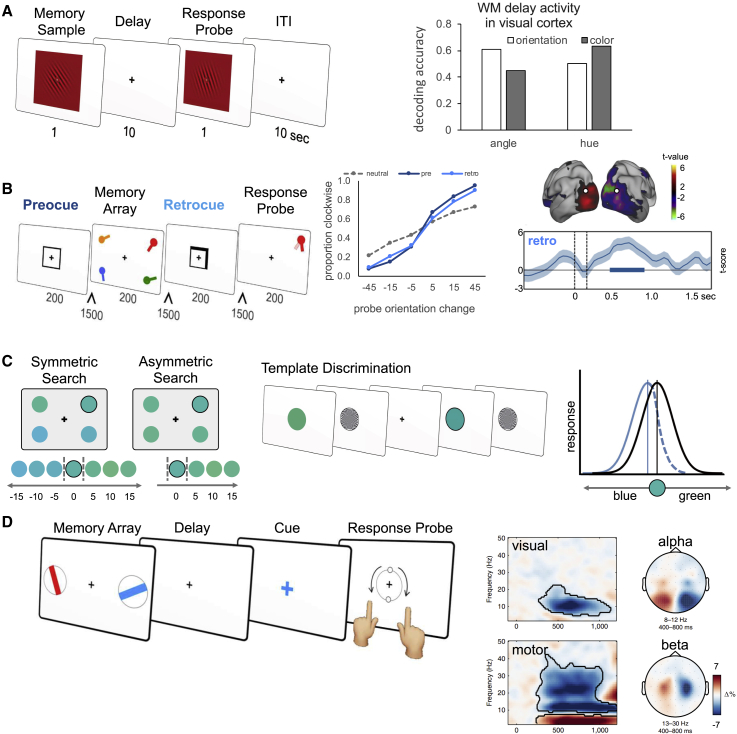
Memory Is Prospective, Representing the Information Most Likely to Be Relevant for Behavior (A) Selective encoding. Serences et al. (2009) used fMRI to show that WM maintains sensory information (color or orientation) that is most relevant to behavior. Decoding patterns of activity in early visual cortex, they found that activity in the memory delay carried orientation angle information when orientation was relevant for future decision-making or color hue information when color was relevant (adapted from Serences et al., 2009). (B) Selective maintenance. Wallis et al. (2015) used MEG to investigate selection of items already in WM. Retro-cues resulted in better memory performance (center panel) and contralateral suppression of alpha power in the visual cortex (right panel; adapted from Wallis et al., 2015). Scale bar represents spectral lateralization (contralateral minus ipsilateral) in normalized units (t-value). (C) Memories can also be distorted to guide behavior. During a visual search task in which distractor stimuli are clustered on one side of the parametric feature space (e.g., color in Yu and Geng, 2019), the search template held in WM becomes distorted away from the veridical target to better separate the target from the competing distractors (adapted from Yu and Geng, 2019). (D) Working memories also code for motor plans when motor affordances are available and can optimize performance. In van Ede et al. (2019), visual stimuli were paired with specific motor plans (left panel), resulting in concurrent visual and motor preparation and their characteristic electrophysiological signatures (right panel; adapted from van Ede et al., 2019). Scale bar represents spectral lateralization (contralateral minus ipsilateral) in percept signal change.

Selectivity in WM is also well documented in the human brain. The CDA marker of WM derived from electroencephalograms (EEGs) shows that encoding can be spatially and item selective (e.g., Vogel and Machizawa, 2004). In addition, selectivity can also occur for relevant features within memory items. Using multivariate pattern analysis (MVPA) to derive population-response properties from fMRI data, Serences et al. (2009) found that decoding during a WM delay depended on the expected demands during recall. Participants performed a delayed matching task on colored gratings and were cued that either color or orientation was relevant to the discrimination. Patterns of activity in the visual cortex selectively maintained the task-relevant feature, consistent with a prospective memory code for guiding future behavior (Figure 3A).

Studies in human participants have also demonstrated that selective contents in WM can be updated flexibly as predictions change about the items or features that will be relevant for task performance (Griffin and Nobre, 2003, Landman et al., 2003). Cues presented during the delay that retrospectively inform participants about the item most likely to be probed (retro-cues) result in significantly better memory performance. fMRI studies show selective maintenance of task-relevant features after informative retro-cues that predict or instruct which stimulus or attributes will be probed (Kuo et al., 2014, LaRocque et al., 2017, Lepsien and Nobre, 2007, Sligte et al., 2009). Human neurophysiological studies also show flexible updating of selective content in WM during maintenance with retro-cues that indicate which item in memory is most likely to be probed (Kuo et al., 2012, Poch et al., 2014, Wallis et al., 2015; Figure 3B). Such updating can also occur in the absence of external cues, when the passage of time is associated with the likely item to be probed, illustrating the flexibility of updating selective WM contents and the role of temporal expectations (van Ede et al., 2017).

From a functional perspective, it is not always optimal simply to maintain a veridical representation of previous input. When making some types of fine-grained delayed discriminations, it can be more advantageous to focus on neural information tuned away from the behaviorally relevant feature to maximize separability from distractor items (Navalpakkam and Itti, 2007). In such tasks, behavioral measures confirm that WM templates are adaptive distortions of the experienced information (Navalpakkam and Itti, 2007, Geng et al., 2017). Application of multivariate methods to derive tuning functions of fMRI voxels confirmed that WM templates in such tasks reflect distortions of the perceived stimulus, thus being adaptive rather than veridical (Scolari et al., 2012). Interestingly, in addition to being shifted, the relevant feature information maintained in the target template can also be sharpened to increase separability from distractor stimuli in the task (Geng et al., 2017, Yu and Geng, 2019; Figure 3C).

These studies clearly reveal the prospective face of WM: content is optimized to the expected demands of future processing. In some cases, the prospective WM representation triggered by a stimulus bears no physical resemblance to it. Rainer et al. (1999) trained monkeys on a paired-associate task that systematically manipulated the relationship between memory items and expected probe stimuli. This revealed a subset of neurons coding for the expected stimulus during the delay period even though it was not actually presented on that trial. This also demonstrates the interaction between WM and LTM.

WM representations also go beyond the experienced stimulus to construct motor representations when specific actions can be anticipated. Most WM tasks tend to probe the perceptual content of the memoranda and typically isolate perceptual qualities of stimuli from particular responses. In everyday life, however, the perceptual content of WM often also carries motor affordances, such as when reaching out and grasping the cup of coffee outside your field of view or repeating a telephone number to a friend. In a recent visual WM task that linked the reporting of particular stimulus orientations to a specific hand, it became clear that motor representations are derived from visually encoded stimuli and that motor information is in a similar state of readiness as visual content when participants are probed (van Ede et al., 2019; Figure 3D).

#### Future-Relevant Content of LTM

The prospective attributes of LTM are much more difficult to study. Except under highly constrained experimental conditions, it is difficult to determine what aspects of experience will prove useful to guide future behavior at distant time points. Nevertheless, the prospective nature of LTM can be gleaned from a few observations. The content of LTM is selective. We better remember items that were relevant during the encoding context (Aly and Turk-Browne, 2017). We retain aspects of stimuli that are useful for adaptive behavior. Although our memories of the details in the appearance of a penny are shockingly poor (Nickerson and Adams, 1979), we readily remember the attributes that distinguish a penny from other coins. Similarly, our well-established difficulty with individuating and remembering faces from other cultures and races may, in part, reflect our focus on discriminant features that are informative within our given cultural context (Rhodes et al., 1989).

### Functional States for Memory-Guided Attention

When considering the prospective nature of memory, studies have typically focused on the task dependency of the representational content (as reviewed above). This information-based perspective provides an important starting point. At minimum, the content of memory provides the basic information needed for adaptive guidance of preparatory attention. However, we need to delve deeper to understand how memories proactively exert their influence in adaptive behavior. Focusing specifically on WM, we consider how mnemonic states functionally contribute to memory-dependent processing of subsequent input.

#### Computation-Specific Codes

**Figure 4:**
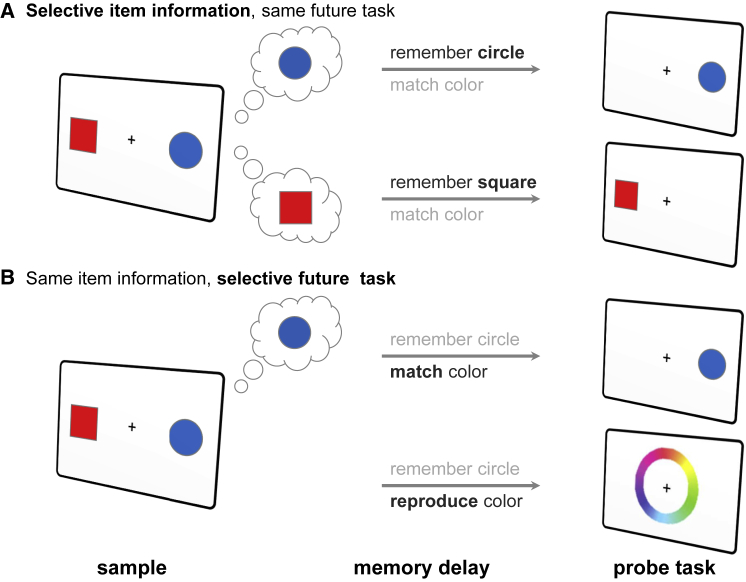
Preparing for Specific Items or Specific Tasks (A) Typically, the prospective nature of WM is studied by varying the type of information that will be probed at the end of the trial. For example, if participants are cued that circles are task-relevant, then they preferentially encode and maintain information corresponding to those items. (B) However, WM tasks can also differ in how the items will be used (e.g., match-to-sample versus reproduction task). From a functional perspective, the neural format can also be adaptive for the type of expected future task.

From a functional perspective, we can distinguish the task relevance of specific types of information from their intended use (Figure 4). For example, tasks may differ in terms of which stimuli are relevant (e.g., squares versus circles in a match-to-sample paradigm), but tasks can also differ in how memories will be used. The same memory item (e.g., red square) could be required for comparing it with a previous item (e.g., color match-to-sample) or for reproducing its exact hue (e.g., continuous report). Under these circumstances, the same information is maintained but for different computational purposes.

Theoretical simulations suggest that WM may be coded differently depending on expected future use. Orhan and Ma (2019) found that a recurrent neural network trained to perform WM exploited a range of different possible coding schemes depending on the precise nature of the memory task. Essentially, the same information content of a visual stimulus (e.g., orientation) was stored differently depending on how it would be interrogated at the end of the delay.

The extant empirical data are also consistent with the theoretical proposal that the format of WM depends on expected use. For example, Warden and Miller (2010) showed that delay-related activity in the PFC differs when the monkey remembers the same stimuli (natural objects) for different types of recall (match judgement versus serial order). Muhle-Karbe et al. (2017) reported similar evidence in humans. Using fMRI, they found that the patterns of neural activity coding for visual stimuli during a memory delay critically depended on how memories were to be used at the end of the trial (implement versus recall).

If computational specificity is functionally relevant to future processing and also inherent to WM, then we might predict that the content of WM always guides attention. The evidence for this hypothesis is mixed. In a series of behavioral and neuroimaging studies, Soto et al. (2008) found that holding an item in WM automatically biased attention to matching items in an unrelated secondary search task. This effect persisted even when the task strongly encouraged participants to mentally separate the WM item from the search task (Soto and Humphreys, 2007). This obligatory link suggests that simply holding something in WM is sufficient for guiding attention. However, it turns out that the story is a bit more complicated. When there is more than one item in the WM set, it is easier to decouple from attentional selection (Downing and Dodds, 2004).

To reconcile the apparent contradiction, Olivers et al. (2011) proposed that one item in WM is in a prioritized state and serves as an attentional template. We term this the “active” item. If only one item is in memory, then it is automatically also the active item guiding visual search. If, however, there are multiple items in WM, then only one item is functionally active, and others act as functionally “latent” items. In the absence of any particular difference in relevance among items, priority could be assigned to any one of them. On average, this dilutes the effect of automatic attentional capture because the critical item only coincides with the active one on some trials. Unless priority is under direct experimental control, it is difficult to disentangle variability in priority state across trials from a diluted effect shared across items.

Active and latent memories can be studied more directly by experimentally manipulating the relative priority of items in WM using retro-cues (Griffin and Nobre, 2003). Behavioral and neural evidence confirmed that participants can flexibly toggle priority between different items in WM, resulting in performance benefits for the currently relevant item. Interestingly, latent items are not forgotten, just temporarily de-prioritized while the active item guides current behavior (Lepsien and Nobre, 2007, Myers et al., 2018, van Ede et al., 2018).

It is tempting to think of retro-cueing simply as an internal spotlight boosting the representational content of cued relative to uncued (and potentially distracting) memoranda. However, we argue that retro-cues may also transform the format of a memorandum, preparing it for future use, such as by placing it in a prioritized state and linking its informational content to future action (Myers et al., 2017). Moreover, a change in priority status need not result in a cost to other information in WM (Myers et al., 2017). When one item is initially prioritized, the performance cost to uncued items can be overcome when there is time or opportunity to reset priorities (Myers et al., 2018, Rerko and Oberauer, 2013, van Ede et al., 2017). Thus, in contrast to the typical consequence of attention (in perception), trade-offs in information quality are not necessary when prioritizing items in WM (Myers et al., 2017). Instead, deficits arise mainly because item-related traces in WM are not currently optimized for readout.

Human neurophysiology also supports the general view that prioritizing an item in WM involves a discrete state transition (Wallis et al., 2015) rather than sustained internal attention. Using magnetoencephalography (MEG), we observed that shifting priority in WM leads to a transient lateralization of posterior alpha power (Wallis et al., 2015; Figure 3B), indexing a punctate process of spatial selection. This contrasts with a sustained pattern of alpha lateralization, as observed during anticipatory attention in perception (Wallis et al., 2015; see also Worden et al., 2000). We interpret this transient response as a discrete process of setting up the most relevant item to guide future target processing.

Recent brain imaging studies also identified different neural correlates of WM depending on current priority status. There is a quantitative difference in decodability between active and latent items. Across multiple experiments, Lewis-Peacock et al. (2012) have shown that active items more readily decoded from patterns of visual activity. Indeed, latent items often have no detectable neural trace even though they remain in WM and are available for future use when they are re-prioritized (Sprague et al., 2016).

These decoding studies show that latent working memories can be nearly impossible to detect in delay period activity. Nevertheless, complementary recent evidence shows that latent memories may be measured using approaches that are sensitive to changes in neural states that might not be reflected in tonic activity. The impulse response approach (Wolff et al., 2015) borrows from the logic of active sonar, in which a well-characterized impulse (“ping”) is emitted toward a hidden landscape, and the contours are inferred from distortions in the reflected signal. In the case of “neural sonar,” a neutral visual stimulus acts as a sensory ping impulse and interacts with the brain state, resulting in a state-dependent impulse response. Changes in the neural landscape can be inferred from distortions in this output response. Importantly, this approach is theoretically sensitive to any change in the functional state of the targeted system. In addition to the manifest delay activity relating to firing in neuronal assemblies, which has been the focus of most WM studies to date, it can also reveal activity-silent neural states, such as temporary changes in synaptic weights within neuronal assemblies (Stokes, 2015).

Using this approach, it has been possible to observe robust memory signals in the impulse response despite dramatic differences in activity-based decoding between active and latent items (Rose et al., 2016, Wolff et al., 2017). This suggests that even functionally latent states have a robust neural signature in silent neural states. Therefore, latent WM is not necessarily just a weaker version of active WM, but, rather, it can be maintained in a qualitatively different neural state. Such a scheme makes sense when one considers that latent memories carry information that may become important down the line and can therefore be just as important to maintain as currently active memories. The important difference is that latent memories should be kept functionally dormant while the active memory is guiding behavior to avoid cross-item interference. Some recent fMRI studies provide initial support for this idea. For example, recent fMRI studies identified different brain areas (Christophel et al., 2017) and different coding schemes within the visual cortex (van Loon et al., 2018) associated with active and latent working memories.

#### How WM States Influence Attention

The classic biased-competition model of attention proposed that perceptual templates in WM bias visual processing to prioritize task-relevant items (Desimone and Duncan, 1995). This idea is grounded in the notion that WM is maintained via persistent activation of sensory-specific neural codes (e.g., Chelazzi et al., 1993), resulting in an elevated baseline for subsequent processing of related input. For example, persistent delay activity associated with remembering the letter X essentially pre-activates the neural code for X, providing a head start for the sensory processing of X (or X-related) stimuli (Desimone and Duncan, 1995).

Indeed, there is extensive evidence for such persistent activity associated with WM and preparatory attention. During WM delays, persistent activity occurs across many brain areas (Christophel et al., 2017), from the visual cortex (Pasternak and Greenlee, 2005) right up to the PFC (Curtis and D’Esposito, 2003). The earliest evidence for WM-related persistent activity was observed in the monkey PFC (e.g., Fuster and Alexander, 1971). WM delay activity is selective for the content of memory; specific cells are more active when their preferred (relative to non-preferred) stimulus is held in mind (e.g., Funahashi et al., 1989, Miller et al., 1996). At the population level, item selectivity gives rise to a population code that can be “decoded” by downstream regions. Similar evidence for item-decodable engrams have also been reported in the parietal cortex (Chafee and Goldman-Rakic, 1998) and in visual areas such as the inferior temporal cortex (Fuster and Jervey, 1981). Correspondingly, brain imaging studies using multivoxel pattern analysis have found stimulus-specific delay activity throughout the human visual system (e.g., Serences et al., 2009) and higher-order brain areas (Ester et al., 2015). This suggests that WM could be a systems-level phenomenon (Christophel et al., 2017), with different areas contributing to distinct but complementary functions (e.g., Dotson et al., 2018).

A very similar profile of activity is observed for preparatory attention. In preparation for visual search guided by a specific object or location in WM, activity in visual areas representing the relevant object (Chelazzi et al., 1993) or location (Luck et al., 1997) is elevated in anticipation of the search array. Human fMRI studies have also reported elevated levels of activity for the spatial location (Kastner et al., 1999) or identity (Stokes et al., 2009) of relevant, anticipated objects based on WM templates. Such findings have supported the influential idea that persistent activity associated with maintenance in WM provides the major neurophysiological mechanism for top-down attentional modulation by effectively biasing subsequent activation of matching sensory input (Desimone and Duncan, 1995). According to a simple baseline shift model, persistent activity for WM becomes preparatory activity for attention, giving relevant information an advantageous head start in competitive sensory processing.

Despite the general appeal of elevated baseline activity linking WM and attention, the specific neurophysiological evidence is not straightforward. Although classic evidence for item-specific delay activity highlighted pre-activation of target items, the actual correspondence between selectivity during the delay and sensory-related responses is more complex (Hayden and Gallant, 2009). A neuron’s stimulus preference during the delay often differs from preferences during stimulus processing in the visual cortex (e.g., Mirabella et al., 2007) as well as the PFC (Stokes et al., 2013, Spaak et al., 2017, Wasmuht et al., 2018). The evidence is also mixed for another core prediction: pre-stimulus activity should directly translate to a corresponding boost in the sensory signal. Although a number of studies found that trial-wise variance in item-specific delay activity correlates with behavioral performance (e.g., Giesbrecht et al., 2006), there is minimal evidence that this is achieved through a corresponding boost to target processing (e.g., Fannon et al., 2008). For example, in classic studies demonstrating anticipatory sustained delay activity (e.g., Chelazzi et al., 1993), the initial neuronal response to the target array is equivalent regardless of whether the target to be selected is effective or ineffective at driving the cell. Only later does neuronal firing settle into a strong or weak response depending on whether the target was effective or ineffective, respectively.

**Figure 5:**
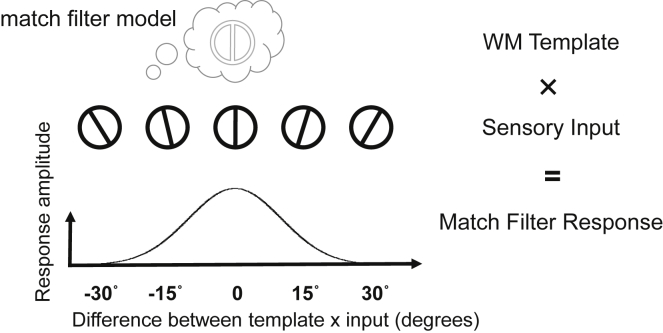
Setting the Initial Condition in WM for Attention Modulation through a Match Filter Model WM establishes a match filter in sensory areas that computes the perceptual similarity between incoming sensory signals and an internal template. A match filter template need not involve pre-activation of the target stimulus. Moreover, the match enhancement effect could serve a general salience cue for capturing attention.

A different but related idea is that WM establishes a match filter in sensory areas that effectively computes the perceptual similarity between incoming sensory signals and an internal template (Hayden and Gallant, 2009, Sugase-Miyamoto et al., 2008, Myers et al., 2015; Figure 5). Theoretically, a match filter template does not depend on pre-activation of target templates but, rather, can be instantiated by more complex activity dynamics (e.g., Machens et al., 2005) or by short-term synaptic plasticity (Sugase-Miyamoto et al., 2008, Myers et al., 2015). This idea can be tested by examining the response to memory-matching stimuli rather than delay activity. For example, in a classic study, Motter (1994) found an enhanced response for stimuli that matched the cued color in V4. Hayden and Gallant (2009) observed a similar enhancement for matching stimuli, which they interpreted as evidence for a match filter mechanism. Sugase-Miyamoto et al. (2008) further demonstrated that the amplitude of match-related activity in single neurons in high-level visual areas co-varied with the amplitude of activity during encoding rather than during delay activity. This pattern was consistent with predictions from their match filter model instantiated with synaptic plasticity. Using MEG in human participants, we showed that perceptual decision-making based on templates held in WM is also consistent with a match filter mechanism (Myers et al., 2015).

Although the match filter model is typically invoked to explain WM decisions (e.g., Hayden and Gallant, 2009, Myers et al., 2015), the match-related enhancement signal could also serve attentional selection, providing a relative boost to target stimuli. Moreover, the same match-related enhancement would also naturally benefit the processing of related features, such as items in the spatial location of the match object. Match-related attentional enhancement is consistent with previous evidence presented above for automatic memory-based capture (e.g., Soto et al., 2008).

#### Setting the Initial Conditions for Memory-Guided Attention

In general terms, WM can be thought of as setting the initial conditions for state-dependent processing (Stokes et al., 2013). The initial condition at the time of target processing is determined by previous input. Differences in stimulus history cause differences in the initial condition, which, in turn, alter the response dynamics of the system during target processing (Remington et al., 2018). These state-dependent dynamics can perform a number of memory-related operations (Buonomano and Maass, 2009), such as reproduce a previous item (Mongillo et al., 2008) and its temporal delay (Wolff et al., 2019), bias the incoming signal for a match-filter response (Sugase-Miyamoto et al., 2008), adaptively distort processing based on off-channel tuning for optimal discrimination performance (Navalpakkam and Itti 2007), and instantiate a temporary decision rule for flexible decision-making (Mante et al., 2013). Importantly, the previous input that defines the initial conditions includes recent stimulation history (e.g., specific items in WM) but also stretches back across timescales (e.g., task instructions and learning form intermediate and LTM).

Mnemonic states could be expressed via elevated patterns of neuronal activity and/or altered patterns of synaptic weights over short and long timescales. In terms of WM specifically, understanding the relative contributions of neural states defined by activity patterns versus activity-silent traces remains an important area for investigation (Constantinidis et al., 2018, Lundqvist et al., 2018). Various accounts propose mappings between different functional and neural states in WM; functionally active WM is expressed as decodable activity, whereas functionally latent states are silent (e.g., Rose et al., 2016, Sprague et al., 2016, Wolff et al., 2017). However, at least theoretically, functionally active or latent memories could be stored in either activity-dependent or silent neural states. We should not conflate the two notions of active.

In summary, a functional perspective re-casts WM as a flexible shift in how the brain prepares to process future information rather than just maintaining a representation of past information. Considered this way, it is the functionality of the neural state that is most important and not merely the decodability of memory content. Decodability is only a minimal requirement for a memory. To understand how memories are stored for future use (recall, attention, or anything else), it is necessary to understand how mnemonic states interact with subsequent input to produce appropriate output. Recent methodological developments provide an expanding toolbox for exploring the functional properties of mnemonic states. For example, impulse perturbations provide a powerful tool for probing the functional state during memory delays (Wolff et al., 2017). Analysis tools for characterizing context-dependent neural dynamics (Remington et al., 2018) will also shed further light on the critical interaction between WM and subsequent processing.

#### LTM Biases

In principle, many of the mechanisms we have considered in the context of how working memories guide future processing may also apply to LTM. It will be important for future studies to determine the extent to which activity-silent LTM representations modulate information processing directly, depending on the overlap between present and past settings and goals. It will be informative to understand under what conditions long-term traces are prioritized and energized to guide adaptive behavior. In the latter context, it will also be interesting to learn whether or when activated LTM traces necessarily operate through functionally active WM states to guide attention (e.g., Atkinson and Shiffrin, 2016).

### Conclusion

We have considered memory through a different temporal lens. Rather than focusing on how memories echo the past, we have considered how we use past experience to anticipate relevant events to unfold. In addition to providing evidence that traces across several timescales premember events proactively and dynamically, we argue that this basic ecological function of memory shapes the content and the format of what is stored, maintained, and accessed. Significant work lies ahead to understand how mnemonic neural substrates facilitate the processing of incoming information to guide adaptive behavior. Looking at memory from this perspective opens many interesting doors for future exploration, such as the types of memory-based modulatory mechanisms, the existence of a common or multiple memory-based priority maps to guide attention, and how memories can be selective and context dependent and yet be used to generalize to novel situations.
